# Leveraging the Medicines for Malaria Venture malaria and pathogen boxes to discover chemical inhibitors of East Coast fever

**DOI:** 10.1016/j.ijpddr.2019.01.002

**Published:** 2019-01-25

**Authors:** James Nyagwange, Elias Awino, Edwin Tijhaar, Nicholas Svitek, Roger Pelle, Vishvanath Nene

**Affiliations:** a International Livestock Research Institute, P. O. Box 30709, Nairobi, Kenya; b Cell Biology and Immunology Group, Wageningen University, the Netherlands; c Biosciences Eastern and Central Africa, International Livestock Research Institute (BecA-ILRI) Hub, P. O. Box 30709, Nairobi, Kenya

**Keywords:** Medicines for malaria venture, Malaria box, East coast fever, Dasatinib, *Theileria parva*

## Abstract

Chemotherapy of East Coast fever, a lymphoproliferative cancer-like disease of cattle causing significant economic losses in Africa, is largely dependent on the use of buparvaquone, a drug that was developed in the late 1980's. The disease is caused by the tick-borne protozoan pathogen *Theileria parva*. Buparvaquone can be used prophylactically and it is also active against tropical theileriosis, caused by the related parasite *Theileria annulata*. Recently, drug resistance was reported in *T. annulata*, and could occur in *T. parva*. Using a ^3^H-thymidine incorporation assay we screened 796 open source compounds from the Medicines for Malaria Venture (MMV) to discover novel chemicals with potential inhibitory activity to *T. parva*. We identified nine malaria box compounds and eight pathogen box compounds that inhibited the proliferation of F100TpM, a *T. parva* infected lymphocyte cell line. However, only two compounds, MMV008212 and MMV688372 represent promising leads with IC_50_ values of 0.78 and 0.61 μM, respectively, and CC_50_ values > 5 μM. The remaining compounds exhibited a high degree of toxicity (CC_50_ values < 1.09 μM) on the proliferation of bovine peripheral blood mononuclear cells stimulated with concanavalin A. We also tested the anti-cancer drug, dasatinib, used in the chemotherapy of some leukemias. Dasatinib was as active and safe as buparvaquone *in vitro*, with an IC_50_ of 5 and 4.2 nM, respectively, and CC_50_ > 10 μM. Our preliminary data suggest that it may be possible to repurpose compounds from the cancer field as well as MMV as novel anti-*T. parva* molecules.

## Highlights

• 17 of 796 MMV compounds inhibited proliferation of *T parva* infected lymphocytes.
• 15 of the 17 hit compounds also inhibited proliferation of control cells.
• MMV008212 and MMV688372 represent promising leads, therapeutic index >8.
• Dasatinib was as active and safe as buparvaquone *in vitro,* therapeutic index >2000.

## Graphical abstract

**Figure:**
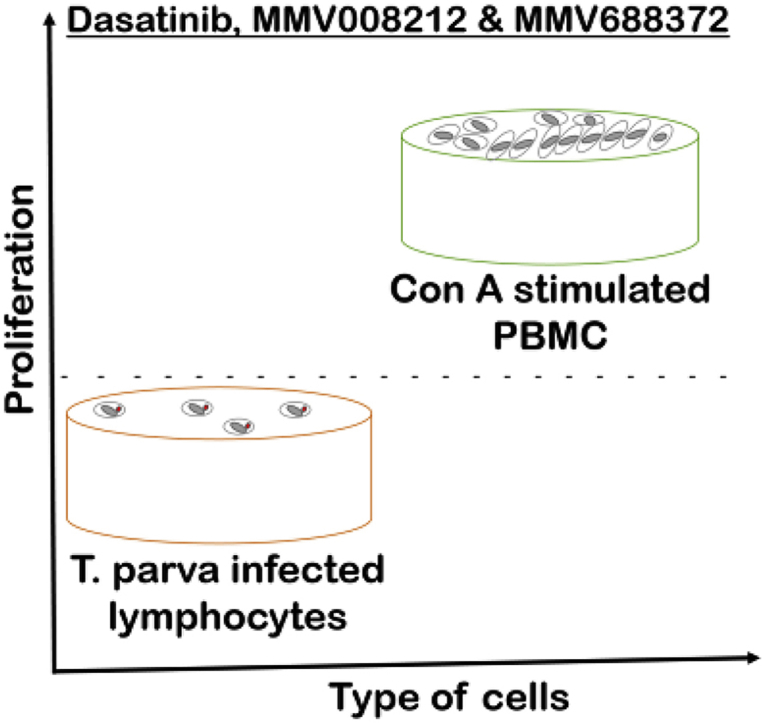

## 1 Introduction

East Coast fever (ECF), a lymphoproliferative disease caused by *Theileria parva* is the most important tick-borne disease in sub-Saharan Africa, killing cattle and causing economic losses (Norval et al., 1992). *Theileria* belongs to the phylum *Apicomplexa*, which contains other parasites responsible for major human and animal diseases, such as malaria, babesiosis, toxoplasmosis and cryptosporidiosis (Morrison, 2009). Considerable progress has been made in developing vaccines for the control of some of these diseases. For example, a live infection and treatment method of immunization is commercially available for the control of ECF (reviewed in Perry, 2016) and tissue-culture vaccines are available for the control of tropical theileriosis caused by *T. annulata* (Brown, 1990). Advanced clinical trials are in progress with a malaria vaccine (Clinical Trials Partnership, 2015). However, drug treatment often remains a frontline method of disease control.

Derivatives of naphthoquinones exhibit significant pharmacological properties and have given rise to development of anti-parasitic drugs, including commercial products to control malaria, e.g., atavaquone, a hydroxy-napthoquinone (Nixon et al., 2013). One of the byproducts of this effort gave rise to development of anti-theilerial drugs, e.g., parvaquone and the improved current drug of choice for treatment of theileriosis, buparvaquone (McHardy et al., 1985). Members of this class of compounds also display activity against other human and animal pathogens, e.g., trypanosomes (Salas et al., 2011). Unfortunately, resistance to atavaquone, an analog of ubiquinone, emerged quite rapidly and drug resistance to atavaquone in *Plasmodium* is associated with mutations in the mitochondrial gene encoding apo-cytochrome b (Vaidya and Mather, 2000). Resistance to other classes of anti-malarial drugs has also emerged, limiting the efficacy of several frontline drugs (Cui et al., 2015). The Medicines for Malaria Venture (MMV) was inaugurated in 1999, with a mandate of developing new anti-malarial drugs, as it was recognized that the pipeline for developing such drugs was small and not an attractive venture for the pharmaceutical industry.

Resistance to buparvaquone in *T. parva* has not been described. However, the recent identification of drug resistance in *T. annulata* (Mhadhbi et al., 2010; Sharifiyazdi et al., 2012) is a cause for concern as it could occur in *T. parva*. As for many tropical diseases, there is no program to develop new anti-theilerial drugs. Although focused on developing novel anti-malarial drugs, MMV has catalyzed neglected disease drug discovery by making available drug-like and probe-like compounds to the scientific community through the malaria box (Van Voorhis et al., 2016) and followed by the pathogen box. A unique aspect of the biology of *T. parva* and *T. annulata* is that schizont-infected leukocytes behave and proliferate like cancer cells *in vitro*, a property that is dependent on parasite viability (reviewed in Dobbelaere and Rottenberg, 2003). These infected cells are the principal cause of disease. We have taken advantage of this phenomenon and the availability of MMV compounds to screen for molecules that inhibit the growth the *T. parva* infected cells.

We screened compounds in both the malaria and pathogen boxes against F100TpM, a bovine lymphocyte cell line infected with the schizont stage of *T. parva* and against bovine peripheral blood mononuclear cells (PBMC) stimulated by concanavalin A (ConA), the latter to determine toxicity on uninfected but proliferating bovine lymphocytes. We identified two compounds with an *in vitro* therapeutic index >5, which could act as starting points for discovery of novel anti-theilerial drugs. In addition, we screened an anti-cancer drug, dasatinib, which is used for treatment of chronic myelogenous leukemia and acute lymphoblastic leukemia as an inhibitor of protein-tyrosine kinases (Steinberg, 2007; Talpaz et al., 2006) as these enzymes are modulated in *T. parva* infected cells (Fich et al., 1998). Dasatinib was found to selectively inhibit F100TpM cells with an *in vitro* therapeutic index >2000.

## 2 Methods

### 2.1 Experimental compounds

The MMV malaria box and pathogen box were obtained from the Medicines for Malaria Venture (MMV, Geneva, Switzerland). Plate mapping and full data on the compounds in the malaria box were accessed at (http://www.mmv.org/research-development/malaria-box-supporting information) and (http://www.pathogenbox.org/) for the pathogen box. All compounds were received in plates at 10 mM stock concentrations and were diluted in 100% DMSO to form copies of each plate containing each compound at 1 mM concentration. From the 1 mM stock plates serial dilutions were made in RPMI 1640 culture media to a 10 μM working stock plates. Buparvaquone (Butalex^®^) was used as a positive control drug and dasatinib was purchased from Selleckchem, USA (cat no. S1021). The molecular weight (MW) and lipophilicity index (ALogP) of active compounds were provided with the data sheets from MMV and are listed in Table 1 and Table 2.

**Table 1:** Malaria box compound inhibitory activity.

Compound	Structure^a^	MW^b^ g/mol	ALogP^c^	IC_50_ (μM) F100TpM	CC_50_ (μM) Con A blasts	TI^d^ (CC_50_/IC_50_)	Activity on other organisms (reference)
MMV008212		280.32	3.59	0.78	>10	>12	*Trypanosoma cruzi, P. berghei* ookinete, *P. falciparum* gametocyte NF54-late stage (Van Voorhis et al., 2016)
MMV498479		245.27	2.32	0.05	0.13	2.6	*T. cruzi* (Van Voorhis et al., 2016)
MMV006455		370.49	3.97	0.84	0.90	1.07	*P. falciparum* respiratory target in early ring stage and gametocyte (Van Voorhis et al., 2016)
MMV665820		293.53	3.13	0.15	0.25	1.67	*Cryptosporidium parvum, Leishmania donovani* axenic amastigotes (Van Voorhis et al., 2016), *T. annulata* (Hostettler et al., 2016)
MMV665841		273.33	3.13	0.36	0.66	1.83	*T. cruzi* and *Wolbachia* (Van Voorhis et al., 2016)
MMV665800		347.84	4.33	0.49	0.53	1.08	*P. falciparum* (inhibitor of ATP4 activity) and *P.berghei* (Van Voorhis et al., 2016)
MMV000356		379.27	4.16	0.87	0.86	0.99	*Leishmania donovani* amastigotes axenic (extracellular) (Van Voorhis et al., 2016)
MMV007363		232.71	3.3	1.93	1.09	0.56	*T. cruzi, Toxoplasma gondii* (Van Voorhis et al., 2016)
MMV007273		480.58	7.24	0.04	0.06	1.5	*P. falciparum* (Van Voorhis et al., 2016)
Buparvaquone (control drug)		326.435	6.45	0.0042	>10	>2380	*Theileria annulata* (Mhadhbi et al., 2010)

a Structure of compound from ChemSpider (http://www.chemspider.com/) or DrugBank (https://www.drugbank.ca/).

b Molecular weight of the compound.

c Lipophilicity index of the compound.

d Therapeutic index (TI) of the compound.

**Table 2:** Pathogen box compounds inhibitory activity.

Compound	Structure^a^	MW^b^ (g/mol)	ALogP^c^	IC_50_ (μM) F100TpM	CC_50_ (μM) Con A blasts	T.I^d^ (CC_50_/IC_50_)	Activity on other organisms (reference)
MMV688372		401.44	3.83	0.61	5.3	8.69	Anti-Trypanosoma (Duffy et al., 2017)
MMV676600		474.55	2.06	0.55	<0.001	<0.001	Anti-Trypanosoma (Duffy et al., 2017)
MMV676602		460.57	3.83	0.35	0.30	0.86	Anti-Trypanosoma (Duffy et al., 2017)
MMV688180		495.43	3.22	0.97	<0.001	<0.001	Anti-Trypanosoma (Duffy et al., 2017)
MMV688271		403.27	3.81	0.59	0.56	0.95	Anti-Trypanosoma (Duffy et al., 2017)
MMV023985		324.38	3.18	0.52	<0.001	<0.001	Anti-malarial, CDPK1 or PK7 proposed targets (Duffy et al., 2017)
MMV003152 (Mebendazole)		295.29	3.06	0.36	<0.001	<0.001	Anti-helminthic (Oxberry et al., 2001)
MMV688978 (Auranofin)		678.484	**-**	0.54	0.40	0.74	Rheumatoid arthritis drug in human (Jeon et al., 2003)

a Structure of compound from ChemSpider (http://www.chemspider.com/) or DrugBank (https://www.drugbank.ca/).

b Molecular weight of the compound.

c Lipophilicity index of the compound.

d Therapeutic index (TI) of the compound.

### 2.2 In vitro cell growth assays

All the MMV compounds were initially tested for inhibitory activity on the growth of F100TpM, a bovine lymphocyte cell line infected by *T. parva*. Cells were seeded in 100 μl RPMI 1640 media at 1 × 10^4^ cells per well in 96 well plates and incubated overnight in 5% CO_2_ at 37 °C before addition of compounds at 1 μM final concentration for 48 h prior to pulsing with ^3^H-thymidine. The IC_50_ of inhibitory compounds was determined by titration of the compound from 0 to 2 μM final concentration. Compounds found to inhibit growth of F100TpM were further screened for non-specific toxicity on the proliferation of uninfected bovine cells stimulated with ConA. Briefly, 1 × 10^4^ PBMCs isolated from uninfected bovine blood by Ficoll-Paque density gradient centrifugation were seeded in 100 μl RPMI 1640 media per well in 96 well plates and cultured overnight in 5% CO_2_ at 37 °C in the presence of ConA (final concentration of 5μg/ml) before addition of compounds from 0 to 10 μM final concentration for 48 h prior to pulsing with ^3^H-thymidine. Due to their high efficacy, lower drug concentrations of buparvaquone and dasatinib were used, 0–0.08 μM final concentration, to determine their IC_50_ and CC_50_ values. All compounds were tested in triplicate.

### 2.3 Thymidine incorporation as a marker for inhibitory activity

The radiolabeled precursor ^3^H-thymidine is incorporated into DNA strands during cell replication and radioisotope incorporation is often used as a measure of cell proliferation. All cells were pulsed with 20 μl of RPMI 1640 media containing ^3^H-thymidine (0.5 μCi) per well and incubated in 5% CO2 at 37 °C for a minimum of 8 h s before harvesting. The cells were then harvested by a Filtermat Harvester (Perkin Elmer) onto Glass fiber matt filters (part No. 6005422) that captures DNA. The filters were then air-dried and placed in Omni Filter cassette and 30 μl of MicroScintTM (Perkin Elmer Cat No 6013611) liquid added, then sealed with TopSeal (Perkin Elmer, Part No 6050195) and loaded on TopCount NXT reader (Perkin Elmer) for determination of incorporation of ^3^H-thymidine.

### 2.4 Calculation of IC_50_ and CC_50_ for bioactive compounds

IC_50_ values were calculated using the Graph Pad Prism 7 software (GraphPad Software, Inc., La Jolla, CA), and average values of radio-isotope incorporation in wells with and without compound on the growth of F100TpM cells. The CC_50_ values were calculated using ^3^H-thymidine incorporation values determined on the growth of PBMC stimulated with ConA. The therapeutic index of a compound was calculated by dividing the CC_50_ value with the IC_50_ value.

## 3 Results and discussion

In a search for new lead anti-theilerial compounds, we screened the open source MMV malaria box and pathogen box using a multi-step process. First, we used compound-induced inhibition of incorporation of ^3^H-thymidine by a *T. parva* infected cell line (F100TpM) at a final concentration of 1 μM as a marker of anti-theilerial activity. Proliferation of infected cells is parasite-dependent and appears to occur through complex manipulation of several host-cell signaling and metabolic pathways (Metheni et al., 2015; Shiels et al., 2006). Only 17 of the 796 compounds were found to exhibit inhibitory activity on F100TpM cell growth. There was little difference in the incorporation of radiolabel in the control and non-inhibitory compound wells indicating that residual levels of DMSO in these wells were not toxic to the cells (data not shown). Second, the IC_50_ of compounds that were active on F100TpM was determined as an indication of *in vitro* potency. Third, to rule out non-specific inhibitory activity on proliferating cells, these compounds were also tested on bovine PBMCs stimulated with ConA and used to calculate CC_50_ values, as an indicator of non-specific *in vitro* cell toxicity. Most *T. parva* infected cells lines are derived by “transformation” of T-lymphocytes by the parasite (Baldwin et al., 1988). Since ConA drives the proliferation of T-lymphocytes (Palacios, 1982) we reasoned that ConA blasts represent a more suitable and stringent cell line control for the *in vitro* assays.

Nine compounds from the malaria box, with IC_50_ values ranging from 0.04 to 1.93 μM (Table 1), and eight compounds from the pathogen box, with IC_50_ values ranging from 0.35 to 0.97 μM (Table 2) inhibited F100TpM proliferation, representing a 2% hit rate. However, the majority of these compounds were highly toxic on the control cell line. Only two compounds, one from each box, MMV008212 and MMV688372, exhibited an *in vitro* therapeutic index of 8 and 10, respectively. An index >5 has been used to determine parasite selectivity of MMV compounds (Duffy et al., 2017). MMV008212 and MMV688372 exhibit an ALogP lipophilicity index of 3.59 and 3.83, respectively. This index is one of several indicators used in compound design of drugs destined for oral or systemic delivery. It describes the partition coefficient between an organic, e.g., octanol, and aqueous phase and, hence, its potential bioavailability as a drug (Comer and Tam, 2001). The structure of the MMV compounds is depicted in Table 1, Table 2. For many compounds the therapeutic index was close to or less than one. In our hands, the positive control drug, buparvaquone had an IC_50_ of 4.2 nM and a CC_50_ > 10 μM, with a therapeutic index of more than 2000. Interestingly, four pathogen box compounds were more inhibitory on the control cell line, than on F100TpM, indicating that there could be differences in the molecular and cellular biology of the two cell lines.

The malaria box compound identified with anti-theilerial activity, MMV008212, also has activities against *Trypanosoma cruzi* (100% growth inhibition at 5 μM), *Plasmodium berghei* ookinete (76% growth inhibition at 10 μM), *P. falciparum* gametocyte NF54-late stage (91% growth inhibition at 5 μM) and *P. falciparum* 3D7 asexual blood stage (98% growth inhibition at 5 μM) (reviewed in Van Voorhis et al., 2016). Cytotoxicity of this compound was observed only in one cell line (U87, 76% cell death at 5 μM) of the nine cell lines tested (Van Voorhis et al., 2016). However, the mode of action of this compound is still unknown. The unsuitable remaining eight malaria box compounds are reported to exhibit varied inhibitory activities on other organisms including *Plasmodium, Toxoplasma, Leishmania, Cryptosporidium* and *Wolbachia* (Table 1). In contrast to our finding, these compounds exhibited less cytotoxicity on the mammalian cell lines and zebrafish embryo that were tested (Van Voorhis et al., 2016).

MV688372, the pathogen box compound identified here with a high therapeutic index against F100TpM cells has anti-trypanosomal activity. This compound is a substituted 2-phenylimidazopyridine with activities against *Trypanosoma brucei brucei* and *T. cruzi* with therapeutic indexes of 230 and 24, respectively, in relation to HEK293 cells (Duffy et al., 2017). The mode of action of MMV688372 is still unclear, but a structurally related molecule, azabenzoxazole, has been identified as a proteasome inhibitor in kinetoplastid parasites (Duffy et al., 2017). Of the remaining seven compounds with a low therapeutic index, five exhibit anti-trypanosomal activity, one has anti-malarial activity and two are reference compounds (Table 2). The latter are interesting components of the pathogen box as mebendazole (MMV003152) is a known anti-helminthic drug that binds tubulin, inhibiting its assembly into microtubules and consequently perturbing the motility, division and the secretion processes of cells (Wampande et al., 2007). In helminths, binding of the drug leads to loss of cytoplasmic microtubules which perturbs glucose intake leading to exhaustion of glycogen reserves and consequent death (Oxberry et al., 2001). The second reference compound, auranofin (MMV688978), is a drug indicated for rheumatoid arthritis and thought to act through inhibition of nuclear factor kappa-B kinase subunit beta (Jeon et al., 2003) and thioredoxin reductase (Rigobello et al., 2005) leading to reduced inflammatory responses and reduced free radical production, respectively. Although both reference compounds demonstrated inhibitory activities on F100TpM with IC50s < 1 μM, their low therapeutic index questions a role for them as anti-theilerial lead compounds.

The malaria box has been screened before for inhibitory activity on *Theileria annulata* infected cell lines and only one inhibitory compound in our assays, MMV665820, was also inhibitory to *T. annulata* cell lines (Hostettler et al., 2016). Although this compound did not impair viability of the human foreskin fibroblast control cells in the *T. annulata* assay, it was eliminated as a lead compound since it failed to lower the relative expression of *T. annulata* gene TaSP by 50%, suggesting that its activity was not parasite specific (Hostettler et al., 2016). In our assay, the therapeutic index for this compound was 1.67 leading to its elimination as a lead compound. The different profile of inhibitory compound activities on these two highly related parasite cell lines illustrates the differences in biology between these parasites and underscores the difficulty associated with discovery and development of anti-theilerial drug(s). We are not aware of a screen of compounds from the pathogen box on *T. annulata* cell lines. Hence, MV688372, should be tested against *T. annulata* cell lines as this might lead to discovery of compound(s) effective in both organisms.

Dasatinib exhibited very potent inhibitory activity on F100TpM cells with IC_50_ (5 nM) and CC_50_ (>10 μM), values similar to buparvaquone, the current drug of choice for chemotherapy of ECF. It will be interesting to test dasatinib activity on *T. annulata* and other transforming *Theileria* pathogen cell lines. Dasatinib is a commercial drug approved and indicated for chronic myelogenous leukemia and acute lymphoblastic leukemia (Steinberg, 2007; Talpaz et al., 2006). It is being considered for treatment of other cancers (Finn et al., 2007; Hochhaus et al., 2008; Johnson et al., 2010). In humans, the drug has multiple targets including tyrosine-protein kinases (Bcr-Abl, Lck and Src family) (Quintas-Cardama et al., 2006), platelet-derived growth factor receptor beta (Chen et al., 2006) and signal transducer and activator of transcription 5B (Nam et al., 2007). Therefore, dasatinib's mode of action on *T. parva* infected lymphocytes could be through one or a combination of the targets, as these cells exhibit cancer-like properties with an up-regulation of growth factor receptors, NF-kB and tyrosine protein kinase cell-signaling pathways, among other cellular changes (Shiels et al., 2006). This raises the possibility that the inhibitory activity of dasatinib on *T. parva* infected cells is via inhibiting a host cellular pathway, rather than directly targeting the pathogen, and opens an additional avenue for drug discovery for the control of ECF.

In conclusion, we have identified two lead MMV compounds one from the malaria box and the other from the pathogen box with a therapeutic index >5 that could form starting points for development of alternative drugs to control ECF, and, possibly other forms of theileriosis. In addition, we have demonstrated a very high therapeutic index of dasatinib on a *T. parva* infected lymphocyte cell line. A large number of related tyrosine kinase inhibitors are available and suggests that these molecules could be explored for their use as repurposed drugs, provided cost is not an inhibitory factor.

## Conflicts of interest

Authors declare no conflict of interest.

## Appendix A Supplementary data

Data Profile Data Profile

Malaria Box 400 compounds Malaria Box 400 compounds
